# Brain structure changes over time in normal and mildly impaired aged persons

**DOI:** 10.3934/Neuroscience.2020009

**Published:** 2020-05-20

**Authors:** Charles D Smith, Linda J Van Eldik, Gregory A Jicha, Frederick A Schmitt, Peter T Nelson, Erin L Abner, Richard J Kryscio, Ronan R Murphy, Anders H Andersen

**Affiliations:** 1 Department of Neurology, University of Kentucky College of Medicine, Lexington, Kentucky, USA; 2 Magnetic Resonance Imaging and Spectroscopy Center, University of Kentucky, Lexington, Kentucky, USA; 3 Alzheimer's Disease Center, Sanders-Brown Center on Aging, University of Kentucky, Lexington, Kentucky, USA; 4 Department of Neuroscience, University of Kentucky, Lexington, Kentucky, USA; 5 Department of Pathology and Laboratory Medicine, University of Kentucky, Lexington, Kentucky, USA; 6 Department of Epidemiology, University of Kentucky, Lexington, Kentucky, USA; 7 Department of Statistics, University of Kentucky, Lexington, Kentucky, USA

**Keywords:** aging, cognition, grey matter loss, CSF expansion, hippocampal volume loss

## Abstract

Structural brain changes in aging are known to occur even in the absence of dementia, but the magnitudes and regions involved vary between studies. To further characterize these changes, we analyzed paired MRI images acquired with identical protocols and scanner over a median 5.8-year interval. The normal study group comprised 78 elders (25M 53F, baseline age range 70–78 years) who underwent an annual standardized expert assessment of cognition and health and who maintained normal cognition for the duration of the study. We found a longitudinal grey matter (GM) loss rate of 2.56 ± 0.07 ml/year (0.20 ± 0.04%/year) and a cerebrospinal fluid (CSF) expansion rate of 2.97 ± 0.07 ml/year (0.22 ± 0.04%/year). Hippocampal volume loss rate was higher than the GM and CSF global rates, 0.0114 ± 0.0004 ml/year (0.49 ± 0.04%/year). Regions of greatest GM loss were posterior inferior frontal lobe, medial parietal lobe and dorsal cerebellum. Rates of GM loss and CSF expansion were on the low end of the range of other published values, perhaps due to the relatively good health of the elder volunteers in this study. An additional smaller group of 6 subjects diagnosed with MCI at baseline were followed as well, and comparisons were made with the normal group in terms of both global and regional GM loss and CSF expansion rates. An increased rate of GM loss was found in the hippocampus bilaterally for the MCI group.

## 1. Introduction

Knowledge of brain structure is an important foundation for understanding and interpreting changes in functional capacity as people age, and for gauging the effects of age-related diseases. Disentangling chronologic structural brain aging effects from those due to common age-associated disease such as Alzheimer's disease (AD) and microvascular brain injury remains a challenge [1]. Despite this challenge, the results of prior structural imaging studies suggest that there is an age related reduction in grey matter (GM) volume and increased ventricular volume even in the absence of significant underlying disease diagnosed at autopsy [2]–[5]. In cognitively normal adults, specific regions of GM volume reduction with age have been associated with corresponding reductions in function. For example, loss in the prefrontal cortex affects working memory performance [6] and loss in medial temporal lobe affects episodic memory [7].

In this study, we characterized longitudinal rates of global and regional cortical GM volume and cerebrospinal fluid (CSF) volume change in a well-characterized cohort of cognitively normal aged adults undergoing a standardized magnetic resonance imaging (MRI) protocol performed twice over an approximately six year interval. The data from this study demonstrates global patterns of GM loss and total (ventricular and sulcal) CSF volume increase in our cohort. A small number of these participants developed cognitive changes over the study interval, and the data presented demonstrates regional differences in GM loss rates in these persons. Additionally, a smaller group of subjects diagnosed with MCI at intake were followed as well for comparison.

## 2. Materials and methods

### 2.1. Participant characteristics

Participants in the current study were followed longitudinally at the University of Kentucky Alzheimer's Disease Center (ADC) as described previously in detail [8]. Each participant underwent annual neurologic and medical examination, neurocognitive testing, and consensus review. A subset of the cohort was recruited to participate in additional MRI studies. Participants provided informed consent under University of Kentucky Medical Institutional Review Board approved procedures. An initial scan was performed on 146 subjects; 84 of these subjects received a repeat scan median 5.8 years later (IQR 5.6–6.0 years), providing scan pairs for analysis. On recruitment, each participant was considered to be cognitively normal, but six had been clinically diagnosed with MCI by the time of the initial scan, leaving a study group of 78 normal controls at baseline.

Diagnosis of Possible and Probable AD used National Institutes of Health-Alzheimer's Disease Research Centers (NIA-ADRC) criteria [9]. MCI diagnostic criteria have undergone changes over time; participants diagnosed as MCI during the observation period would be classified currently as late amnestic MCI [10],[11]. Other degenerative illnesses were diagnosed according to published literature standards, and individuals with those diagnoses were not eligible for this study.

### 2.2. Scan protocol

A standardized MRI imaging protocol was performed on each participant using the same research Siemens Magnetom 1.5 T MRI scanner, and the same circularly-polarized quadrature head coil was used for both baseline and repeat image sessions. T1-weighted images (repeat time TR 400 ms, echo time TE 12 ms, 2 acquisitions) were acquired as 2 sets, each with 3 mm slices, 1.25 × 0.94 mm in plane resolution, slice gap 3 mm interleaved to eliminate inter-slice interference. The two separate sets with 6 mm spacing of slice centers were offset 3 mm from each other so the sets themselves could be interleaved to form a volumetric image with 3 mm resolution in the z-direction. T2-weighted double-echo images (TR 4000, TE 20 & 80 ms) were acquired with the same resolution and slice positions as the T1 images (intrinsically registered). FLAIR and gradient-echo sequences were also acquired but not used in the present study.

### 2.3. Image processing protocol

T1, T2 (TE 80 ms) and proton density (PD; TE 20 ms) images were resampled at 1 mm isotropic voxel dimensions, mutually registered and N4-corrected [12]. They were then segmented in SPM-12 (Wellcome Trust Center for Neuroimaging; http://www.fil.ion.ucl.ac.uk/spm) with a multispectral approach using a custom template warped and applied to the native-space images to create separate grey matter (GM), white matter (WM) and cerebrospinal fluid (CSF) masks and tissue probability maps.

Because WM signal is often heterogeneous in this age group, WM tissue was modeled initially as comprising two tissues in SPM-12, each corresponding to WM in the template, giving improved representation [13]. There was a rough correspondence between regions of normal-appearing WM and regions of periventricular hyperintensities in the two segments, but not consistently enough to use them separately; nonetheless the sum better represented total WM than when modeled as a single tissue in SPM-12.

For analysis of volumes, segmented images were created in native space, warped to the template and modulated within the unified segmentation regime of SPM-12. Estimates of GM and CSF volume were computed for each scan by summing each segmented image and multiplying by voxel size. Total intracranial volume (TIV) was calculated in native space by summing baseline GM, WM and CSF segmented image masks. TIV calculated from the second image set in each participant was very similar to the first (adjusted r^2^ = 0.95, p < 0.0001). An independently determined TIV from skull-stripping the T1-weighted images in FSL (https://fsl.fmrib.ox.ac.uk/fsl/fslwiki) was used to test reliability of this measurement; the two estimates of TIV from the same subject scan were highly correlated (adjusted r^2^ = 0.97, p < 0.0001).

**Figure 1.**
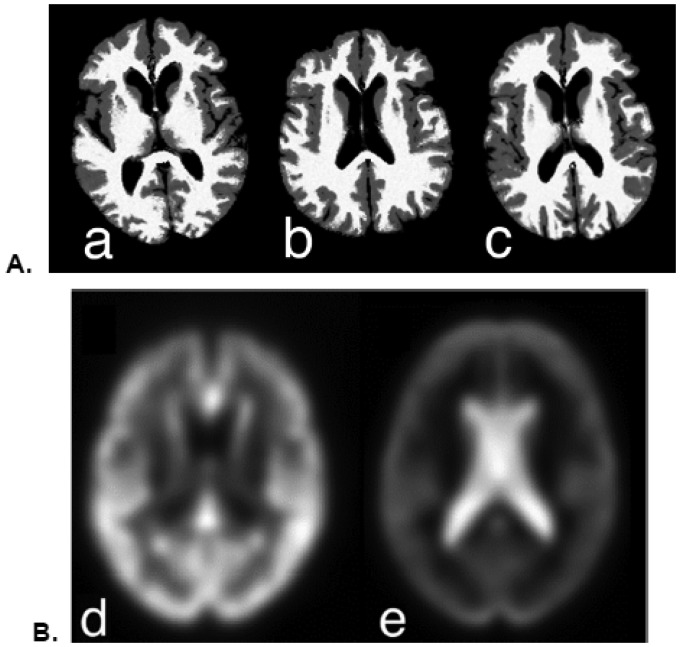
A. Composite scans used for paired longitudinal registration. Composite images have high T1-like contrast derived by combining GM, WM and CSF segmentation probability maps in native space (see text). Initial (a) and repeat (b) scans were often acquired at different orientations. The average image (c) represents the symmetric transformation of a & b into a common image space. B. Averaged shrinkage rate (d) and expansion rate (e) across all 84 subjects, demonstrating widespread GM loss in cortical and subcortical regions (d), and expansion in ventricular and sulcal CSF (e).

Initial longitudinal registration of the original T1-weighted images was inconsistent because of relatively poor grey-white contrast. To overcome this problem, a composite brain image was created from the segmented images using the formula: 1*WM+0.5*GM+0.15*CSF for each subject, where WM, GM and CSF represent the separate probability maps for the respective tissues. This procedure yielded a pair of T1-weighted-like images with consistent contrast for each participant (Figure 1A). Gaussian background noise in phase quadrature and yielding magnitude image intensity values with a Rician distribution was added to the composite to stabilize the background deformation estimates [14],[15]. Actual noise levels were estimated individually from the air background in the subject's T1-weighted images. Paired longitudinal registration using the Ashburner and Ridgway method [16] in SPM-12 was performed on the composite native-space images, producing an average image together with estimates of the divergence of the initial velocity of the flow field (dv) and volume change from the Jacobian transform (jd). The dv images were divided into positive (denoted by the term “expansion rate”) and negative (denoted “shrinkage rate”) images by thresholding at ± 0.001 respectively; negative images were standardized by taking the absolute value (to make all values positive but still representing magnitude of volume change rate). These images and the average images were then normalized to the template space. Smoothed normalized expansion and shrinkage rate images were summed for each subject as an overall estimate of change for inclusion in global rate change models. Spatial maps of averages across subjects in template space were computed as well from these images (Figure 1B). These images represent the divergence of the initial velocity of the flow field used to model decreases in GM and increases in CSF between baseline and repeat scans, and not GM and CSF volume differences themselves. The mean warped average grey matter density image was created as an underlay for displaying results of the image analysis.

### 2.4. Image analysis of regional changes

These analyses were undertaken to demonstrate regional differences between expansion and contraction rates (comparing initial and subsequent MRI results) and between regional GM volumes according to diagnosis. Image analyses were performed in SPM-12 using a flexible factor design, using paired scans as the within subject factor for volume analysis and diagnosis at either the initial or follow-up scan period as the between subject factor in all analyses. Covariates in all analyses were age at first scan, education, gender, years between first and second scan, and total intracranial volume (TIV). Because baseline and repeat scans differed in coverage of the lower half of the cerebellum and brainstem, this region was masked out from the analysis to remove any spurious differences. Significance threshold was p = 10^−5^ (family-wise t-value 5.1).

### 2.5. Models of global structural changes

Standard least squares models of overall non-localized initial volume change rates (expansion and shrinkage rates) incorporated the diagnosis of the subjects at baseline and 5.8 years later (where the diagnosis may have changed), and overall average per-subject change rates calculated from each subject's corresponding images. Covariates in all analyses were age at first scan, education, gender, years between first and second scan, and TIV. Gender by diagnosis interaction terms were included in these models.

### 2.6. Models of hippocampal structural changes

A standard least-squares model for hippocampal volume loss rate was constructed by extracting regional hippocampal shrinkage rate from normalized dv-negative images using the AAL template. Hippocampal volumes extracted from the modulated warped GM images were similar using the ICBM template (adjusted r^2^ = 0.97). The same covariates from the global change models were used, with the addition of each participant's overall dv-negative rate to adjust for global brain shrinkage.

A repeated measures model was constructed for regional hippocampal volumes extracted using the AAL template applied to normalized baseline and repeat scans (rather than loss rates). The respective inverse transformations yield individual volume measures in native space for analysis. The same covariates used in the global change models were used, plus total GM volume at baseline for each participant as an additional covariate.

## 3. Results

Table 1 provides demographic and diagnostic information for the participants analyzed in the current study. At baseline, there were 78 cognitively normal subjects and 6 with MCI. The MCI group was older than the normal group, and had evidence of mild global cognitive change (mean MMSE = 27.0) and a moderate deficit in delayed verbal memory (mean word list delayed recall = 4/10). At the time of the repeat scan 5.8 years after the baseline scan, all 6 of the participants with MCI had been diagnosed with Probable AD, and one additional participant who was normal at baseline had been diagnosed with Probable AD. Additionally, 8 of the baseline normal subjects had received an MCI diagnosis. These incident MCI subjects had the same median MMSE score as the prevalent MCI subjects (MCI diagnosed at baseline), but had higher List Learning (p = 0.01), and List Learning Delay (p = 0.02) scores than their baseline MCI counterparts.

**Table 1.** Demographic and testing results at the time of the initial and repeat scans (median 5.8 years later). Values are median [IQR].

	Baseline			Follow-up		
	Normal (N = 78)	MCI* (N = 6)	AD	Normal (N = 69)	Incident MCI (N = 8)	AD (N = 7)
**Age, years**	73.4	81.9	-	78.8	81.7	85.9^§^
	[66.9–78.4]	[77.4–87.8]		[73.0–83.5]	[73.3–89.3]	[82.2–93.0]
**Gender F/M**	53/25	4/2	-	47/22	5/3	5/2
**Education**	16	15.5	-	16	16	16
	[15–18]	[12.8–16.5]		[15–18]	[12.3–18]	[13–18]
**MMSE**	30	27.0	-	29.0^%^	27.0	19
	[29–30]	[26.8–28.3]		[29–30]	[26–29.8]	[5–22]
**LM-II**	36	19.0	-	36.0^%^	27	7.5
	[31–38]	[11.3–25.3]		[32–39]	13–28]	[3.3–21.3]
**LM%**	98	46.0	-	98^%^	84	4.5
	[90.5–99]	[9.8–74]		[92–99]	[12–86]	[1.8–61.5]
**List Learn**	24	16.5	-	25	20^¶^	18
	[22–26]	[14.5–20]		[22–27]	[18–22]	[16.5–18]
**List Delay**	9	4.0	-	9	6^¶^	3
	[8–9]	[1.5–4.5]		[8–10]	[1–8]	[2–4]

*MCI different from normal baseline on all covariates except gender & education; Wilcoxon rank sum test, p < 0.01; § Older than Normal & MCI; % All three groups different, maximum p< 0.02; ¶ Different from normal but not AD, maximum p < 0.01; Wilcoxon multiple comparison.

Global mean shrinkage rates and expansion rates for the 69 participants who were normal at both the initial and repeat scan were analyzed by one-sample t-tests (without covariates, using no change or zero shrinkage/expansion as the null hypothesis) in a preliminary analysis to gauge the strength and consistency of these changes in either direction. Shrinkage was associated primarily with GM and expansion primarily with ventricular and sulcal CSF (cf. Figure 1B). Overall the GM shrinkage rate was 2.53 ± 0.07 ml/ year (t = 40.0, p < 0.0001), and CSF expansion rate was 2.96 ± 0.07 ml/year (t = 45.6, p < 0.0001). The more inclusive covariate analysis models are detailed below.

**Figure 2.**
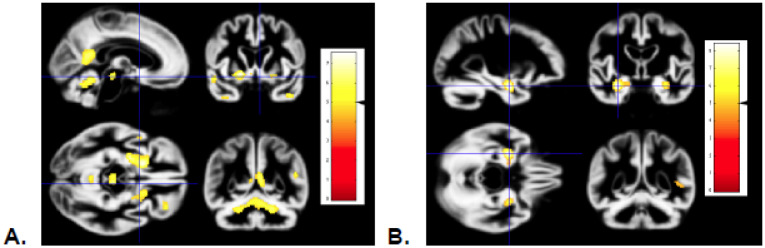
A. Map of decreases in regional GM volume over 5.8 years in 69 subjects normal at both time points. Regional volume loss is seen in ventral posterior frontal lobe, temporal pole, dorsal cerebellum and midbrain grey matter. Significance threshold is p = 0.00001; Family-wise error correction threshold was t = 5.1, indicated by the black arrowhead on the scale shown on the left. B. Increases in regional grey matter volume loss rates in baseline-diagnosed MCI compared to subjects diagnosed as normal at both baseline and repeat study (5.8 years later). Anterior hippocampus demonstrates significant excess volume loss during the MCI to AD transition (threshold p = 0.00001).

Images demonstrating regional GM shrinkage, adjusted for age at first scan, education, gender, years between first and second scan, and TIV are shown in Figure 2A. These maps represent decreases in regional GM volume over 5.8 years in 69 subjects who remained cognitively normal at both time points. The maps demonstrate bilateral decreases in ventral posterior frontal lobe, ventral anterior temporal lobe, lateral superior temporal lobe, insula, dorsal vermis and paravermian region of the cerebellum, midbrain GM, and in left retrosplenial cortex. Regional volume loss in medial temporal lobe characteristic of early AD (hippocampus and entorhinal region) was not detected [17].

Figure 2B illustrates increases in regional GM volume loss rates in 6 baseline-diagnosed MCI who transitioned to AD compared to 69 subjects diagnosed as normal at both baseline and repeat study (5.8 years later). Loss rates were greatest in hippocampal, lateral temporal and parietal regions, including the posterior cingulate. In this comparison, anterior hippocampus particularly demonstrates significant volume loss during the MCI to AD transition. A similar pattern of GM loss rate was seen with patients diagnosed with Probable AD at the time of repeat scan (not shown), which was expected, because 6 of these 7 participants were the same individuals diagnosed with MCI at baseline. The remaining participant transitioned from normal to Probable AD in the interval between scans. In that same interval, the number of normal subjects for comparison decreased from 78 to 69 because of 8 conversions from normal to MCI and one to AD.

### 3.1. Models of structural changes

Table 2A shows the results of the analysis of global expansion rate at baseline and at the time of the repeat scan. Only participants with MCI at baseline and AD diagnosis at repeat scan demonstrated significantly increased expansion rate compared to the group of normal controls. The incident MCI cases did not show significant expansion. Global contraction (shrinkage) rate demonstrated the identical pattern (Table 2B). None of the covariates demonstrated significant associations.

**Table 2A.** Overall brain initial expansion rate adjusted for covariates (adjusted volume increase rate), primarily associated with sulcal and ventricular CSF. Scores are t-values; exceptions labeled by F value. Covariates not shown in the table are age, interscan interval, education, gender, gender by diagnosis interaction, and TIV; effects associated with these covariates were not significant.

	Diagnosis at Baseline	Diagnosis at Follow-up
Overall Expansion ml/year		
Normal (N)	2.97 ± 0.07 (78)	2.96 ± 0.07 (69)
AD (N)	-	**3.75 ± 0.24^#^** (7)**^§^**
MCI (N)	3.66* ± 0.25 (6)	2.95 ± 0.19 (8)
**Results in Model**		
Diagnosis	**T = −2.64/p = 0.01**	**F = 5.1/p = 0.01**

*post hoc p = 0.01 compared to control; # post hoc p = 0.002; § 2 of these 7 subjects were diagnosed MCI at baseline, and 5 were normal at baseline.

**Table 2B.** Overall brain initial shrinkage rate adjusted for covariates (adjusted volume decrease rate), primarily associated with GM. Covariates were the same as in 2A.

	Diagnosis at Baseline	Diagnosis at Follow-up
Overall Contraction ml/year		
Normal (N)	2.56 ± 0.07 (78)	2.53 ± 0.07 (69)
AD (N)	-	**3.45 ± 0.24^#^** (7) **^§^**
MCI (N)	3.32 ± 0.26* (6)	2.67 ± 0.19 (8)
**Results in Model**		
Diagnosis	**T = −2.81/p = 0.01**	**F = 6.3/p = 0.0003**

*post hoc p = 0.01 vs. normal control; #post hoc AD vs Normal p = 0.01. ^§^ 2 of these 7 subjects were diagnosed MCI at baseline, and 5 were normal at baseline.

Analyses of left and right hippocampal shrinkage rates were performed individually. Global shrinkage rate was highly correlated with the hippocampal shrinkage rate as expected (p < 0.0001; Table 3). However, the hippocampal shrinkage rate remained significantly elevated in the model (p-value range 0.0003 to < 0.0001), indicating an independent regional contribution to this rate by diagnosis.

**Table 3.** Hippocampal shrinkage rates by hemisphere and diagnoses at baseline and follow-up.

	Diagnosis at Baseline		Diagnosis at Follow-up	
Hippocampal Contraction ml/year	Left	Right	Left	Right
Normal (N)	0.0115 ± 0.0004 (78)	0.0107 ± 0.0004 (78)	0.0124 ± 0.0004 (69)	0.0108 ± 0.0004 (69)
AD (N)	-	**-**	**0.015# ± 0.002** (7)	**0.019# ± 0.002** (7)
MCI (N)	0.020** ± 0.002 (6)	0.020** ± 0.001 (6)	**0.016# ± 0.001** (8)	0.010 ±0.001 (8)
Results in Model**				
Gender	T = −1.23/p = 0.22	**T = −2.27/p = 0.03**	**T = 2.38/p = 0.02**	T = −1.93/p = 0.06
Gender x Diagnosis	T = 1.65/p = 0.10	**T = 2.67/p = 0.01**	**F = 7.8/ p = 0.001**	**F = 4.79/p = 0.01**
Total dv-negative rate	**T = 5.81/p < 0.0001**	**T=5.51/p<0.0001**	**F=7.1/ p=0.01**	**F=24.6/ p<0.0001**
Diagnosis	**T = −4.84/p < 0001**	**T =−5.17/ p < 0.0001**	**F = 5.7/ p = 0.005**	**F = 10.9/ p < 0.0001**

x: post-hoc p-value < 0.0001; #post-hoc p-value < 0.05; ** Age, interscan interval, education, and TIV were not significant in the model.

Analyses of hippocampal volume were performed using the baseline diagnosis (Table 4A) and the diagnosis at the time of repeat scan (Table 4B). Volumes were labeled in template space using the AAL atlas and (inverse) transformed to native space for quantification. Using the baseline diagnosis to classify subjects, the model was significant for TIV as expected (p < 0.001 for both right and left hippocampal volume), since hippocampal volume scales with TIV. Right hippocampal volume was greater than left in all diagnosis groups. At baseline diagnosis there was no difference in mean hippocampal volume in normal or MCI subjects. However, there was a significant decline in both right and left hippocampal volume over time in MCI but not normal subjects.

**Table 4A.** Hippocampal volumes at baseline and at repeat scan (baseline + 5.8 years) for subjects classified using the baseline diagnosis (78 normal controls and 6 MCI). Adjustment variables age, interscan interval, gender, gender by diagnosis, and total GM volume, and education were not significant in the model (between subjects). Within subjects, only the time x diagnosis effect was significant.

	Hippocampal Volume	
Hippocampal Volume, ml	Left	Right
Normal		
Baseline	2.26 ± 0.02	2.72 ± 0.02
Repeat	2.23 ± 0.02	2.70 ± 0.02
MCI		
Baseline	2.20 ±0.02	2.72 ± 0.02
Repeat	2.05 ±0.03	2.55 ± 0.03
**Results in Model**		
Between Subjects		
TIV	**F = 23.9/p < 0.0001**	**F = 27.7/p < 0.0001**
Within Subjects		
Time x diagnosis	**F = 12.0/p = 0.0009**	**F = 9.9/0.002**

x: post-hoc p-value < 0.0001.

**Table 4B.** Hippocampal volumes at baseline and at repeat scan (baseline + 5.8 years) for subjects classified using the diagnosis current at the time of the repeat scan (69 normal controls, 8 MCI and 7 AD). Six of the 7 patients diagnosed with AD at the repeat scan were diagnosed with MCI at baseline; all MCI patients at the repeat scan were normal at baseline. Age, interscan interval, education, gender, gender by diagnosis interaction, and total GM volume were not significant between subjects. Only the time by diagnosis interaction was significant within subjects.

	Hippocampal Volume	
Hippocampal Volume, ml	Left	Right
Normal		
Baseline	2.27 ± 0.02	2.74 ± 0.02
Repeat	2.24 ± 0.02	2.71 ± 0.02
MCI*^1^		
Baseline	2.12 ± 0.02	2.60 ± 0.02
Repeat	2.14 ± 0.02	2.60 ± 0.02
AD*^2^		
Baseline	2.20 ± 0.02	2.73 ± 0.02
Repeat	2.05 ± 0.03	2.56 ± 0.03
**Results in Model**		
Between Subjects		
TIV	**F = 26.2/p < 0.0001**	**F = 29.0/p < 0.0001**
Diagnosis	**F = 4.36/p = 0.02**	F = 1.91/p = 0.16
Within Subjects		
Time x diagnosis	F = 8.4/p = 0.0005*^3^	F= 5.6/0.006*^4^

*^1^ post hoc Normal vs MCI F = 5.73, p = 0.02; *^2^ post hoc Normal vs AD F = 3.81, p = 0.05; *^3^ post hoc Normal vs AD F = 12.3, p = 0.0008; Normal vs MCI F = 3.20, p = 0.08; *^4^ post hoc Normal vs MCI F = 0.8, p = 0.37; Normal vs AD F = 9.8, p = 0.003. x: post-hoc p-value < 0.0001.

## 4. Discussion

In this study of carefully characterized and longitudinally followed elderly research participants, there was significant GM shrinkage and CSF expansion in cognitively normal participants. We found a baseline global GM loss rate of 2.56 ml/year, 7% greater compared with the baseline cross sectional loss estimate of 2.4 ml/year [18]. The GM loss rate for participants who were cognitively normal both at baseline and at follow up was 2.53 ml/year, minimally different.

Our GM loss rate relative to total intracranial volume (0.20%/year) is comparable to the 0.24%/year GM loss observed by Allen et al. in 87 subjects aged 22–88 years [19], although there is a range reported between 0.13 and 0.9% per year [20]–[23]. An annual 0.2% global (whole brain) loss was estimated from 56 longitudinal MRI studies of aging that also included younger subjects, but is lower than the 0.5%/year loss estimate for normal persons over the age of 60 years [24],[25]. These estimates include WM and GM together, and thus might be different from GM alone. However, our CSF expansion rate relative to total intracranial volume (0.22% per year) is only slightly larger than shrinkage rate (0.20% per year), similar to the cross-sectional results of Good et al. [22]. Our results matching GM shrinkage and CSF expansion rates seem more consistent than in studies where a mismatch exists between whole brain shrinkage and CSF expansion [23],[26]. These differences may be due to technique, e.g., ventricular rather than total CSF was measured, or inclusion of WM.

Longitudinal studies have generally shown GM loss and CSF volume increases in normal subjects [23],[24], but some have suggested GM losses in particular do not occur in normal aging or are limited, attributing such changes to underlying medical conditions such as diabetes or hypertension [1],[27],[28]. In this study, we defined normal elders as being judged cognitively normal during a clinical interview by a study physician using the Clinical Dementia Rating Scale and a neurological examination, supported by normal scores on a standard battery of cognitive tests without significant change over the interval between scans [8]. Individuals who did not meet these criteria were analyzed separately.

Longitudinal estimates of volume change rates in one large study were approximately 40% greater than in cross-sectional study of the same subjects compared to the 7% difference we found [29] emphasizing there may be large differences between the two determinations in certain cohorts. Inadequate modeling of trajectory effects may cause discrepancies, particularly in studies across a wide age range [30]. We examined subjects in an age range (70–89) and time interval (approximately 6 years) where substantial curvature in rate versus age is unlikely. We also used a deformation-based technique for longitudinal estimation rather than a volumetric approach that may also account for part of the difference [16].

A survey of other studies indicates important complexity that depends in part on the characteristics of the research cohort being studied. For example, a recent study demonstrated similar rates of CSF expansion (0.14%/year) and GM loss (0.14%/year) with an inverted U-shaped rate versus age curve for WM but no overall loss with age [20]. Other studies have shown no overall WM loss with age and similarity between CSF expansion and GM loss rates [22], In contrast, there also has been reported evidence of complex changes in WM volume with an early increase before age 50 followed by a variable and perhaps accelerating decline in later years [19],[31], particularly in subjects with a high cerebrovascular disease burden [1],[32]. Since cerebrovascular disease is heterogeneous, and related to environmental factors, but is practically universal among the elderly according to pathologic studies [33], cohort variability due to underlying age-associated pathologies may therefore be a major determinant of differences in outcomes of MRI studies.

Regional GM losses with aging have been observed in the frontal lobes, specifically dorsolateral prefrontal cortex and insula [31], but others have suggested relative preservation of the very same subregions [3],[20]. In our subjects we found the greatest GM losses in posteroinferior frontal lobe, posteromedial parietal lobe and dorsal cerebellum (Figure 2), which is similar to the pattern demonstrated in a recent study by Bagarinao et al. [20]. Note that our analysis did not include the lower half of the cerebellum, so we cannot comment on that region. Dorsal cerebellar GM losses have been reported in other studies [3],[20],[34]. Selective frontal lobe volume losses were reported in participants in the population-based Framingham Heart Study [32]. Taken together, these findings suggest that, as in reports of WM volume change with age, variability in underlying pathologies and/or cohort characteristics may lead to differences in rate measurements. Our study participants are highly educated (median 16 years) and motivated volunteers, and may represent a healthier part of the elder population spectrum [8].

The hippocampal region demonstrated shrinkage in normal elders similarly to global GM, consistent with some prior studies [19],[31],[35],[36] but not with the relative limbic preservation found in others [37]. We found higher shrinkage rates in hippocampus after adjusting for global shrinkage rates (0.5%/year versus global 0.2%/year), comparable to Morra et al. (0.66%/year; [35]), but there is a wide reported range for normal subjects, in some cases over 1%/year [21],[36],[38],[39].

Hippocampal atrophy and loss rates in MCI have been found to be greater than in cognitively normal subjects, and still greater in Probable AD subjects [31],[35],[38],[40]–[42]. We found medial temporal atrophy in MCI subjects that broadened to include posterior cingulate and lateral temporal regions as patients progressed from MCI to Probable AD over approximately 6 years (Figure 2). Because of the relatively low number of MCI subjects in this study (6 at baseline and 8 incident at follow up), sensitivity for regional losses is low. However, the finding provides evidence of the face validity of our regional detection methods, because it confirmed previously known focal grey matter losses in hippocampus and not in unexpected regions.

## 5. Conclusion

Strengths of our study include a longitudinally followed and well characterized group of elders who had expert evaluation of their cognitive, neurologic and medical status using a standardized evaluation procedure. Participants underwent imaging with the identical protocol and MRI scanner at both time points, overcoming potential technical difficulties in longitudinal quantitative image analysis due to acquisition and scanner differences [43]. There are some important caveats that should be emphasized, however. The volunteers in the study were mostly highly-educated Caucasians and represent the relatively healthy part of the spectrum of aging, and while that may have reduced variability in the findings, the results should not be generalized to the population at large where the prevalence of cerebrovascular disease in particular is likely higher. Also, because we had only two scans available in each subject, higher order modeling of our longitudinal data was not possible, so we may not have captured accelerations or other changes in rate over time as a function of age. Additionally, the relatively large image slice thickness of 3 mm compared to 3D T1 acquisitions at isotropic 1 mm resolution may have affected our ability to detect more subtle brain changes. However, this limitation may have been compensated by the higher accuracy in the segmentation afforded by the use of multispectral T1, PD and T2-weighed image sets.

We conclude that despite a small but inexorable measurable loss of brain GM and compensating increased CSF, brain health may be preserved for many years, in some cases well into the 80s in our study group.
